# Stabilization of tryptophan hydroxylase 2 by l‐phenylalanine‐induced dimerization

**DOI:** 10.1002/2211-5463.12100

**Published:** 2016-08-22

**Authors:** Kasper D. Tidemand, Hans E. M. Christensen, Niclas Hoeck, Pernille Harris, Jane Boesen, Günther H. Peters

**Affiliations:** ^1^ Department of ChemistryTechnical University of DenmarkKongens LyngbyDenmark

**Keywords:** analytical size exclusion chromatography, differential scanning fluorimetry, enzyme characterization, oligomerization, protein purification

## Abstract

Tryptophan hydroxylase 2 (TPH2) catalyses the initial and rate‐limiting step in the biosynthesis of serotonin, which is associated with a variety of disorders such as depression, obsessive compulsive disorder, and schizophrenia. Full‐length TPH2 is poorly characterized due to low purification quantities caused by its inherent instability. Three truncated variants of human TPH2 (rc*h* TPH2; regulatory and catalytic domain, NΔ47‐rc*h* TPH2; truncation of 47 residues in the N terminus of rc*h* TPH2, and c*h* TPH2; catalytic domain) were expressed, purified, and examined for changes in transition temperature, inactivation rate, and oligomeric state. c*h* TPH2 displayed 14‐ and 11‐fold higher half‐lives compared to rc*h* TPH2 and NΔ47‐rc*h* TPH2, respectively. Differential scanning calorimetry experiments demonstrated that this is caused by premature unfolding of the less stable regulatory domain. By differential scanning fluorimetry, the unfolding transitions of rc*h* TPH2 and NΔ47‐rc*h* TPH2 are found to shift from polyphasic to apparent two‐state by the addition of l‐Trp or l‐Phe. Analytical gel filtration revealed that rc*h* TPH2 and NΔ47‐rc*h* TPH2 reside in a monomer–dimer equilibrium which is significantly shifted toward dimer in the presence of l‐Phe. The dimerizing effect induced by l‐Phe is accompanied by a stabilizing effect, which resulted in a threefold increase in half‐lives of rc*h* TPH2 and NΔ47‐rc*h* TPH2. Addition of l‐Phe to the purification buffer significantly increases the purification yields, which will facilitate characterization of *h*TPH2.

**Abbreviations**
5‐HT: 5‐hydroxytryptamin (serotonin)
5‐HTP: 5‐hydroxytryptophan
AAAH: aromatic amino acid hydroxylase
ACT: aspartate kinase, chorismate mutase and TyrA
BH_4_: tetrahydrobiopterin
c*h*TPH2: catalytic domain of human tryptophan hydroxylase
DSC: differential scanning calorimetry
DSF: differential scanning fluorimetry
HEPES: 4‐(2‐hydroxyethyl)‐1‐piperazineethanesulfonic acid
l‐Phe: l‐phenylalanine
l‐Trp: l‐tryptophan
MBP: maltose binding protein
PAH: phenylalanine hydroxylase
rc*h*TPH2: regulatory and catalytic domains of human tryptophan hydroxylase 2
*rn*PAH: *Rattus norvegicus* phenylalanine hydroxylase
SEC: size exclusion chromatography
TH: tyrosine hydroxylase
TPH: tryptophan hydroxylase

Tryptophan hydroxylase (TPH) catalyses the rate‐limiting reaction in the biosynthesis of the hormone and neurotransmitter serotonin (5‐HT). TPH uses the cofactor Fe^2+^ and the cosubstrates O_2_ and tetrahydrobiopterin (BH_4_) to generate 5‐hydroxytryptophan (5‐HTP) by hydroxylation of l‐tryptophan (l‐Trp). 5‐HTP is enzymatically converted to 5‐HT by aromatic amino acid decarboxylase 1, 2. TPH exists in two isoforms, where isoform 1 (TPH1) is mainly responsible for catalyzing the rate‐limiting step in the biosynthesis of peripheral serotonin, and isoform 2 (TPH2) primarily is associated with the biosynthesis of neuronal serotonin 1. The regulation of serotonin levels is involved in various physiological and psychiatric disorders such as irritable bowel syndrome, depression, obsessive compulsive disorder, and schizophrenia 3. Of these, depression is associated with decreased levels of neuronal serotonin, whereas some gastrointestinal disorders are associated with increased peripheral serotonin levels 4, 5, 6. The serotonergic systems are therefore important targets in treatment of these disorders 7, which makes both TPH isoforms important enzymes to characterize.

Along with phenylalanine hydroxylase (PAH) and tyrosine hydroxylase (TH), TPH forms an enzyme subfamily of iron(II)‐containing mono‐oxygenases collectively referred to as the aromatic amino acid hydroxylases (AAAHs). Various truncated variants of all three AAAHs have been structurally characterized 8, 9, 10, 11, 12. For *h*TPH, the catalytic cores of both isoforms have been structurally and enzymatically characterized 13, 14, 15 due to successful purification strategies 16, 17.

All enzymes in the AAAH family form tetramers through dimers of dimers 18. Each monomeric subunit consists of an N‐terminal regulatory domain, a highly conserved catalytic domain, and a C‐terminal tetramerization domain 19. The N‐terminal domains of PAH and TH contain a characteristic ACT fold motif 12, 20. From sequence analysis, TPH has also been proposed to contain an ACT domain 21. Truncations of the regulatory domains have been found not to change the tetrameric assembly of TPH1 or TPH2 1, 22. In contrast, when the C‐terminal tetramerization domain is also removed, monomeric species are observed 1. Deletions from the C‐terminal domain of rabbit TPH1 have been demonstrated to change the macromolecular structure from a tetramer to predominantly monomeric form without compromising catalytic activity 23, 24.

The available crystal structures of TPH1 only comprise the catalytic domain, while the crystal structure of TPH2 includes both the catalytic and the tetramerization domains 13, 14, 25, 26, 27. The lack of structural information of the regulatory domain is partly due to the great instability of full‐length TPH 16, 28. In TPH, the regulatory domain, in particular, has been found to cause instability and therefore limiting the quantities purified from *Escherichia coli* expression systems 1, 16. The regulatory domain of TPH2 has an additional 46 residues compared to TPH1, which are partly responsible for the limited purification quantities 29. The fundamental role of the regulatory domain and the additional residues in the terminus is, however, poorly understood, as characterization is hampered by the limited purification yields. Therefore, structural insight is obtained from the crystal structure of rat phenylalanine hydroxylase (*rn*PAH) (PDB ID: 1PHZ – Clustal Omega: 35% sequence identity in the regulatory domain and 57% total sequence identity) which comprises the catalytic and the regulatory domains. This structure lacks interpretable electron density in the first 18 residues of the N terminus, suggesting that this region is flexible 20. The flexibility of the N terminus has also been confirmed by an NMR study 30. By alignment of the sequences of *rn*PAH and *h*TPH2 (Fig. S1), it is found that residues 20 to 47 of *h*TPH2 align with the mobile 18 residues of *rn*PAH, which suggests that these residues of *h*TPH2 are mobile and might cause instability and insolubility. Therefore, an NΔ47‐rc*h*TPH2 variant was expressed, purified, and characterized.

The current study sheds light on the influence of the regulatory domain on macromolecular structure and stability of TPH2 by characterizing three truncated variants (sequences shown in Supporting information); c*h*TPH2 (catalytic domain), rc*h*TPH2 (regulatory and catalytic domains), and NΔ47‐rc*h*TPH2 (47 residue truncation in the N‐terminal domain). The tetramerization domain (residue 460–490) was removed to investigate the role of the regulatory domain when TPH2 was not in a tetramer. As TPH2 is poorly characterized due to low stability, additional efforts were made to identify ligands that could increase the stability, and hence purification yield, of TPH2.

## Results and Discussion

### Differential scanning fluorimetry

**Figure 1:**
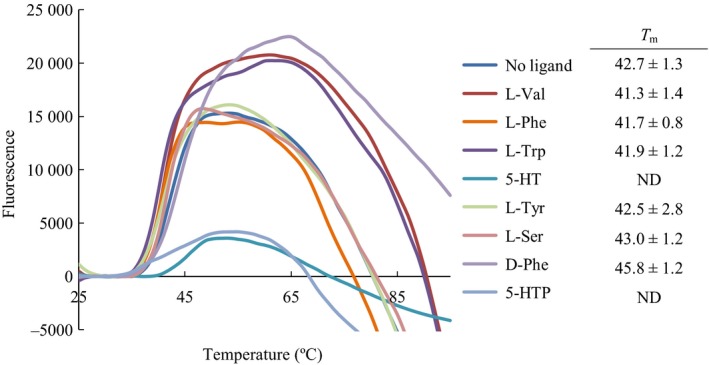
Baseline corrected differential scanning fluorimetry (DSF)– ligand screen on c*h* TPH2 (enzyme concentration of 1 μm). Representative examples of the highest concentration of the ligands in the screen (1.5 mm for tyrosine and 10 mm for all other ligands). To the right *T*_m_ values (*n* = 3) of c*h* TPH2 with the respective ligands are listed (ND, Not detectable).

Very little is known about the regulation and structure of TPH as purification of this enzyme results in limited quantities. The presence of the regulatory domain is known to cause low stability and solubility 1. To overcome this problem, substrates for the AAAH family (l‐phenylalanine, l‐tryptophan, and l‐tyrosine), other ligands known to bind certain ACT domains (l‐valine 31 and l‐serine 32), as well as d‐phenylalanine, 5‐HTP, and 5‐HT were assayed for changes in the thermal unfolding of the TPH2 variants. The ligands were screened in a broad concentration range (0.1 μm–10 mm). For c*h*TPH2, an almost ideal two‐state unfolding behavior was observed, Fig. 1. All ligands, except 5‐HTP and 5‐HT, were found to have no significant effect on the unfolding or *T*_m_ value of c*h*TPH2, Fig. 1. 5‐HTP and 5‐HT appear to change the unfolding of c*h*TPH2 from two‐state to continuous; hence, no *T*_m_ values could be obtained at high ligand concentration.

**Figure 2:**
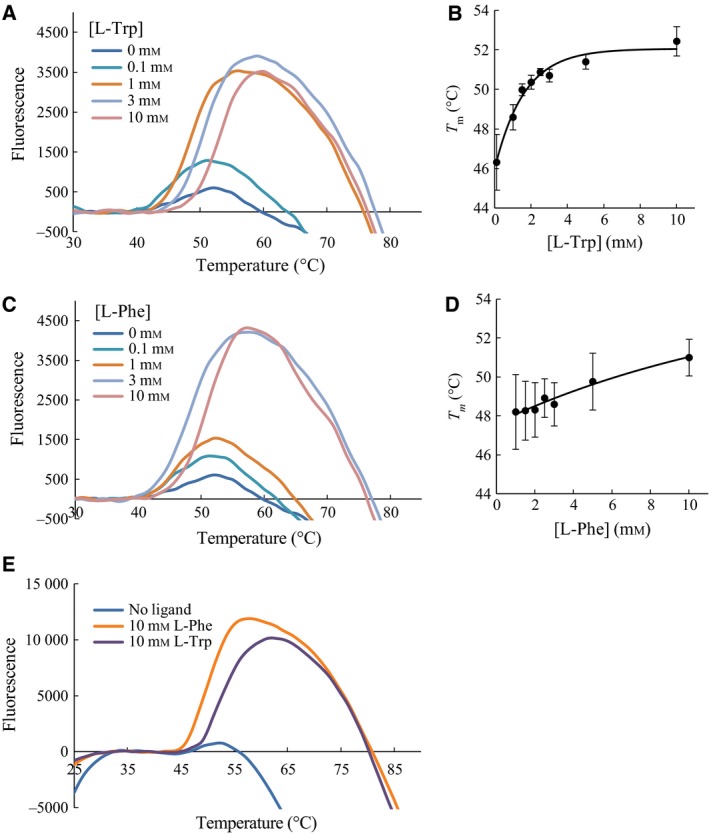
Representative baseline corrected differential scanning calorimetry curves of rc*h* TPH2 or NΔ47‐rc*h* TPH2 (1 μm). (A) DSF curves of rc*h* TPH2 with different l‐Trp concentration. (B) *T*_m_ values with increasing l‐Trp concentration for rc*h* TPH2 with an exponential fit. (C) DSF curves of rc*h* TPH2 with different l‐Phe concentration. (D) *T*_m_ values with increasing l‐Phe concentration for rc*h* TPH2 with an exponential fit. (E) DSF of NΔ47‐rc*h* TPH2 curves in the absence of ligand, presence of 10 mm l‐Phe, or presence of 10 mm l‐Trp.

Containing the regulatory domain, rc*h*TPH2, gave rise to inconsistent and polyphasic unfolding curves from which no *T*_m_ values could be obtained (Fig. S2). The ligand screen showed that l‐Trp and l‐Phe induced unfolding transitions with apparent two‐state unfolding in a concentration‐dependent manner (Fig. 2), which were accompanied by an increase in transition temperatures. The apparent two‐state unfolding was gradually acquired with increasing ligand concentration, and robust apparent two‐state unfolding was obtained at l‐Trp (Fig. 2A) and l‐Phe (Fig. 2C) concentrations of 1.0 mm and 3.0 mm, respectively. At 0.1 mm l‐Trp, rc*h*TPH2 displayed a transition temperature of 46.3 ± 1.4 °C. This was increased to 52.4 ± 0.7 °C by the addition of 10.0 mm l‐Trp. The exponential fit in Fig. 2B shows that saturation has been reached at 10 mm l‐Trp. The transition temperature of rc*h*TPH2 was increased from 48.2 ± 1.9 to 51.0 ± 0.9 °C by increasing the l‐Phe concentration from 1.0 mm to 10.0 mm (Fig. 2D). Similarly, a study by Gersting *et al*. 33 utilizing differential scanning fluorimetry (DSF) found that 1 mm l‐Phe increases the transition temperature of PAH from 47.5 to 50.9 °C. None of the other ligands, including d‐Phe, were able to induce this change in unfolding (data not shown). The same stabilizing trend for l‐Phe and l‐Trp was observed for NΔ47‐rc*h*TPH2, Fig. 2E.

**Figure 3:**
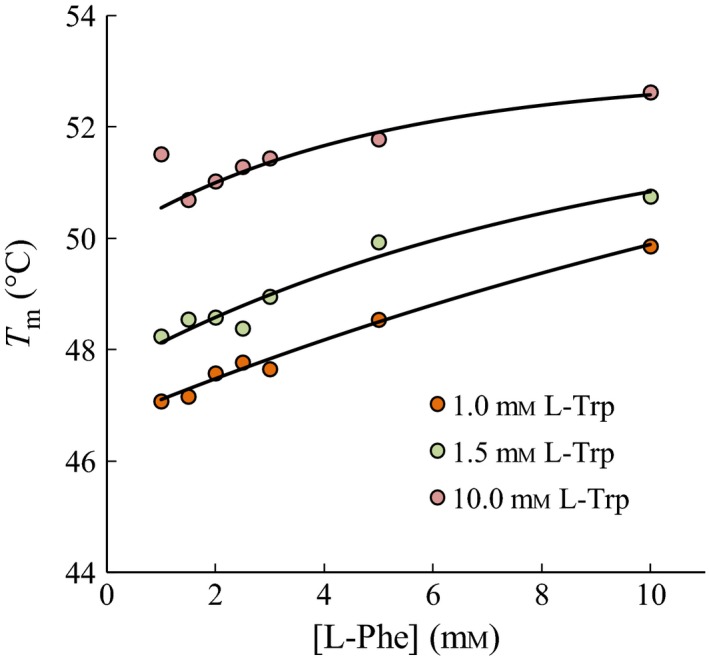
Transition temperatures (*n* = 3–4) obtained from DSF experiments of rc*h* TPH2 with the addition of l‐Trp and l‐Phe. Average standard deviation in each point is 0.8 °C. Curves represent exponential fit of *T*_m_ values as a function of [l‐Phe] at constant [l‐Trp]. Data for the lowest concentration of l‐Phe at 10 mm l‐Trp were omitted from the fit.

As both l‐Trp and l‐Phe gave rise to increased transition temperatures, a combination of the compounds was analyzed. Figure 3 shows that increasing the concentration of either l‐Trp or l‐Phe in the presence of the other compound results in increased *T*_m_ values, suggesting that l‐Trp and l‐Phe are able to increase the transition temperature in an additive fashion. The additive effect is supported by the increase in *T*_m_ values with increasing l‐Phe concentration observed at saturated concentration of l‐Trp (10 mm in Fig. 3).

### Analytical gel filtration

**Figure 4:**
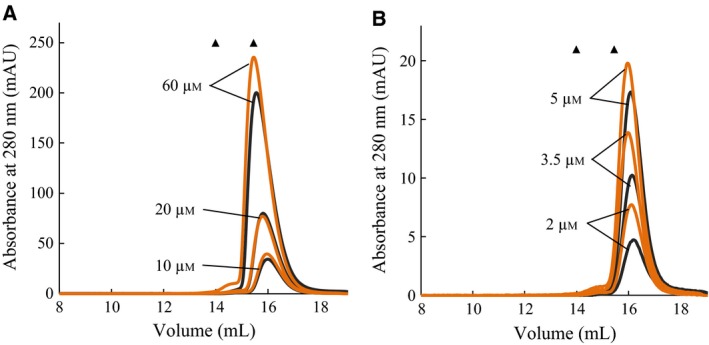
Size exclusion chromatography of c*h* TPH2 with no ligand (black lines) and with the addition of 3 mm l‐phenylalanine (orange lines). The markers (▲) indicate the expected elution volumes of a dimer and a monomer, respectively. Protein loading concentrations are indicated in the chromatograms. (A) High loading concentration range. (B) Low loading concentration range. AU, absorption units.

It has previously been shown in DSF assays that under stabilizing conditions multi‐component complexes can change from polyphasic to almost two‐state unfolding and that this is indicative of monodispersity and stability 34. This was investigated in the case of l‐Phe and l‐Trp as unfolding of rc*h*TPH2 and NΔ47‐rc*h*TPH2 was shifted toward two‐state with the addition of these compounds. The oligomeric states of the TPH2 variants were analyzed utilizing analytical size exclusion chromatography (SEC). In consensus with the findings of D'Sa *et al*. 22, c*h*TPH2 was found to elute at a volume corresponding to the molecular weight of a monomer (36.2 kDa) 35. In the loading concentration range of 2 to 60 μm, c*h*TPH2 was found only to reside as a monomer, Fig. 4. Additionally, 3 mm l‐Phe did not induce any change in the elution pattern of the monodisperse solution complementing the observations from the DSF experiments.

**Figure 5:**
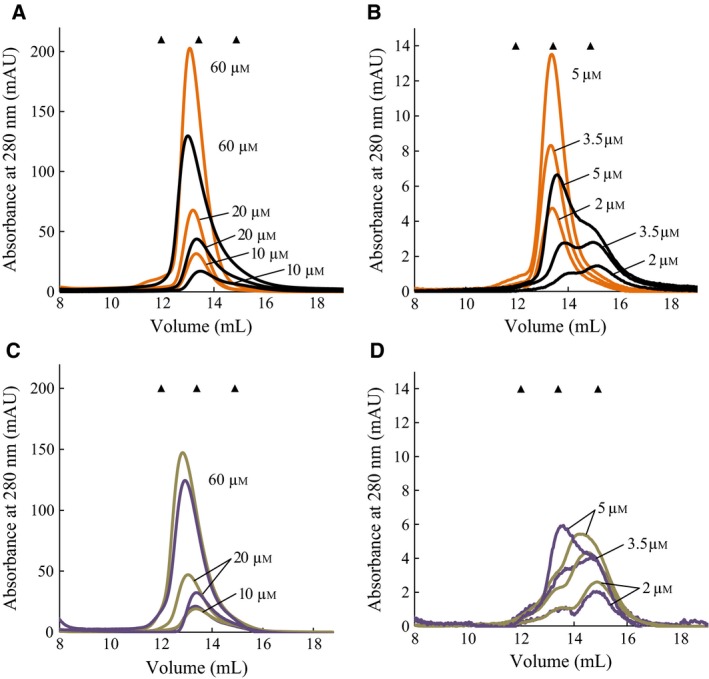
Size exclusion chromatography of NΔ47‐rc*h* TPH2. (A, B) SEC without added l‐Phe (black lines) and with 3 mm l‐Phe (orange lines). A: High loading concentration range. B: Low loading concentration range. (C, D) SEC with the addition of 3 mm d‐Phe (beige lines) or 0.5 mm l‐Trp (purple lines). C: High loading concentration range. (D) Low loading concentration range. The markers (▲) indicate the expected elution volume of (from left to right) a tetramer, a dimer, and a monomer, respectively. Protein loading concentrations are indicated in the chromatograms.

**Figure 6:**
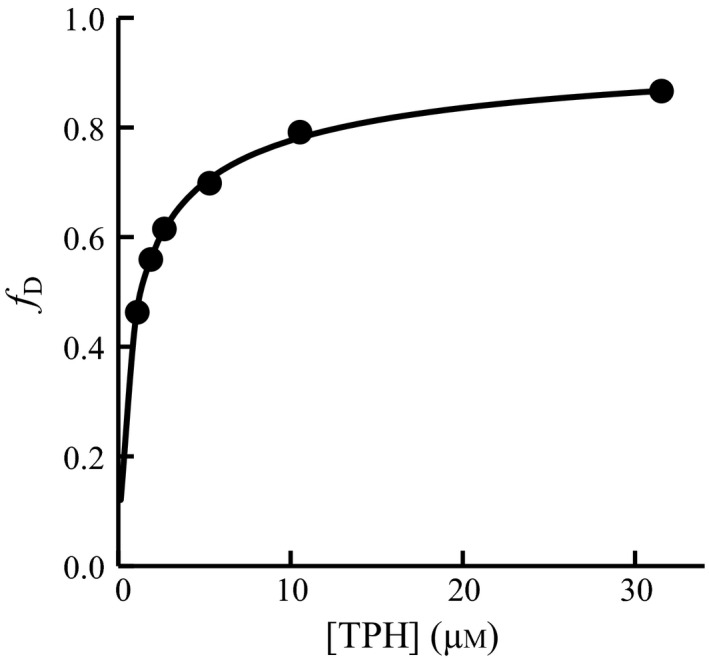
Fraction of dimer as a function of NΔ47‐rc*h* TPH2 concentration (loading concentration divided by dilution factor). The solid line represents the nonlinear regression of the data using equation (11) given in Materials and methods.

A study by Mockus *et al*. 23 on rabbit TPH1 has demonstrated that truncation of the C‐terminal tetramerization domain resulted in a disruption of the tetrameric assembly. This was also found to be the case for *h*TPH2, as SEC of NΔ47‐rc*h*TPH2 yielded two overlapping peaks with elution volumes corresponding to molecular weights of a monomer (47.4 kDa) and a dimer (94.9 kDa) (Fig. 5). The gradual shift of the elution peaks from a monomer to a dimer shows that NΔ47‐rc*h*TPH2 is found to be in a concentration‐dependent monomer–dimer equilibrium. Within experimental error, the peak widths at half height were constant over the range of TPH concentrations, and the peak heights were also found to be related directly to the concentration of TPH injected. The dilution factor was found to be 1.90 ± 0.13 and constant within the elution range of the TPH variants. From the SEC results, the equilibrium constant, *K*_d_, of the dissociation of a dimer into monomers was calculated (equation (7) in Materials and methods). A *K*_d_ value of 1.3 ± 0.1 μm was found based on the six concentrations of NΔ47‐rc*h*TPH2 in the absence of l‐Phe. This value is lower than the dissociation constant of 46 ± 35 μm determined for the dimerization of the regulatory domain of PAH 36. A plot of fraction dimer, *f*_D_, as a function of NΔ47‐rc*h*TPH2 concentration gave a hyperbolic‐like curve as expected for a monomer–dimer equilibrium, Fig. 6.

The difference in oligomeric states of c*h*TPH2 and rc*h*TPH2/NΔ47‐rc*h*TPH2 shows that dimerization is caused by the regulatory domain. This finding is supported by a model structure proposed by Jiang *et al*. 37 in which the regulatory domain of one monomer forms intersubunit interactions to an adjacent monomer. The involvement of the regulatory domain in structural assembly is further supported by results from Yohrling *et al*. 38 on rabbit TPH1, where NΔ41 and NΔ90 truncations resulted in monomers indicating that the regulatory domain is involved in the formation of the tetrameric assembly. D'Sa *et al*. 22, however, found that the tetrameric assembly was retained upon truncation of the regulatory domain of human TPH2. Collectively, this might suggest that both the C‐terminal tetramerization domain and the N‐terminal regulatory domain contribute to the formation of a stable tetramer.

**Figure 7:**
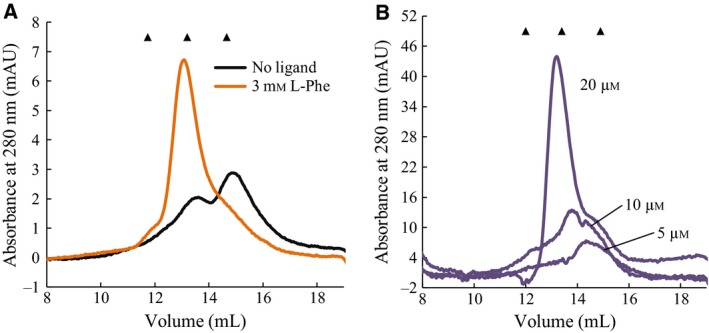
(A) Size exclusion chromatography of rc*h* TPH2 (loading concentration of 2 μm), with no ligand (black line) and with the addition of 3 mm l‐Phe (orange line). (B) Size exclusion chromatography of NΔ47‐rc*h* TPH2, with the addition of 1 mm l‐Trp. Protein loading concentrations are indicated in the chromatogram. The markers (▲) indicate the expected elution volumes of a tetramer, a dimer, and a monomer, respectively.

The effect of l‐Phe on the monomer–dimer equilibrium was analyzed by performing SEC on NΔ47‐rc*h*TPH2 samples with and without the addition of l‐Phe. In Fig. 5, the black curves represent elution of NΔ47‐rc*h*TPH2 without the addition of l‐Phe, and the orange curves represent the elution of protein when the sample and running buffer contained 3 mm l‐Phe. It is evident that l‐Phe shifts the equilibrium toward dimer. At a loading concentration of 2 μm NΔ47‐rc*h*TPH2, the addition of l‐Phe was found to shift the equilibrium from predominately monomer to almost exclusively dimer. The same shift in monomer–dimer equilibrium was observed for rc*h*TPH2 (Fig. 7A). This result correlates well with the observations in the DSF experiments, where the unfolding curves change from polyphasic toward two‐state with the addition of l‐Phe.

The influence of l‐Trp and d‐Phe on the monomer–dimer equilibrium was additionally analyzed to investigate the specificity of l‐Phe. Figure 5C,D shows the monomer–dimer distribution of NΔ47‐rc*h*TPH2 with 0.5 mm l‐Trp or 3 mm d‐Phe in a protein concentrations range from 2 to 60 μm. From the SEC results in Fig. 5, dimerization *K*_d_ values of 1.6 ± 0.7 μm and 1.8 ± 0.9 μm were determined in the presence of l‐Trp and d‐Phe, respectively. 3 mm d‐Phe or 0.5 mm l‐Trp does, therefore, not change the monomer–dimer equilibrium constant. At a TPH2 loading concentration of 2 μm, it is evident by comparison of Fig. 5B,D that neither the addition of 0.5 mm l‐Trp nor 3 mm d‐Phe induced the shift in the monomer–dimer equilibrium toward dimer as observed for l‐Phe. l‐Trp absorption introduced high background noise and was hence only added to concentrations of 0.5 and 1.0 mm. As a consequence of the background noise, chromatograms of NΔ47‐rc*h*TPH2 with loading concentrations below 5 μm could not be obtained at 1.0 mm l‐Trp. However, at low protein concentrations (5–20 μm), the monomer–dimer equilibrium does not seem to be influenced by 1.0 mm l‐Trp as monomer is present (Fig. 7B), which is not observed in the presence of L‐Phe (Fig. 5A,B). The addition of 1 mm l‐Trp is compared with 3 mm l‐Phe as these concentrations were found in the DSF experiments to change the unfolding from polyphasic to apparent two‐state (Fig. 2A,C). The fact that l‐Trp did not induce the same shift in equilibrium suggests that the stabilization observed in the DSF experiments occurs through a different mechanism than for l‐Phe. Furthermore, l‐Phe binding is found to be specific as d‐Phe did not induce dimerization. That L‐Trp does not induce dimerization in *rch*TPH2 extends recent results obtained by Patel *et al*., who showed that among the regulatory domains of TH, PAH, and TPH1, only PAH dimerizes in the presence of its natural substrate 39.

### Thermal inactivation

**Figure 8:**
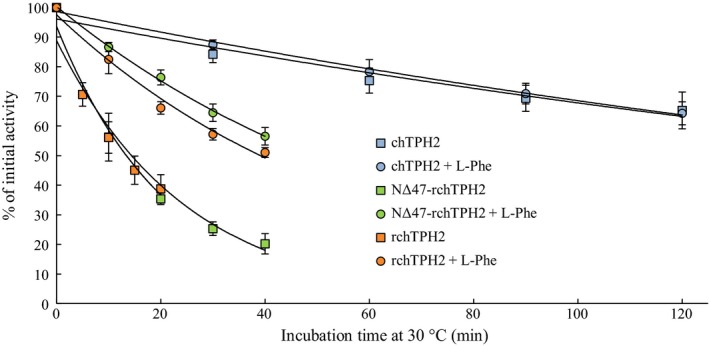
Heat inactivation of hTPH2 variants. Enzyme samples (5 μm) were incubated at 30 °C, and residual activity in % of initial activity at *t*_0_ was assayed in time intervals. The curves are fit to the mean of three independent measurements performed on each variant. Squares and circles present heat inactivation in the absence or presence of 3 mm l‐Phe, respectively.

To investigate if the dimerizing effect had an impact on TPH2 stability, rates of inactivation at 30 °C in the presence or absence of 3 mm l‐Phe were examined. The data were fitted with one‐exponential decay curves, and the rate constants, *k*, were calculated using an exponential function, *E*_*t*_ = *E*_0_e^−*k*t^, where *E*_0_ is the initial enzyme activity, and *E*_*t*_ is the activity after time *t* at 30 °C (Fig. 8). From the decay rate constants, half‐lives (*t*_½_) of the *h*TPH2 variants were calculated using *t*_½_ = ln(2)/*k*. At 30 °C and a concentration of 5 μm, rc*h*TPH2, and NΔ47‐rc*h*TPH2 displayed *t*_½_ values of only 15 ± 2 and 18 ± 3 min, respectively. Hence, truncation of the N terminus slightly increased the *t*_½_ value (*P* = 0.18, *T*‐test). With a *t*_½_ value of 203 ± 40 min, c*h*TPH2 displayed a 14‐fold higher half‐life compared to rc*h*TPH2, which is in agreement with the results of Carkaci‐Salli *et al*. 1 and confirms that the presence of the regulatory domain causes significant destabilization. With addition of 3 mm l‐Phe, *t*_½_ values of 41 ± 3, 49 ± 5, and 193 ± 29 min were obtained for rc*h*TPH2, NΔ47‐rc*h*TPH2, and c*h*TPH2, respectively. Variants containing the regulatory domain displayed nearly 3‐fold increase in half‐lives whereas c*h*TPH2 was not significantly influenced by l‐Phe.

That the stabilizing effect is exclusively observed for rc*h*TPH2 and NΔ47‐rc*h*TPH2 implies that l‐Phe is stabilizing through binding to the regulatory domain. Further, as l‐Phe induces a stabilizing effect in both rc*h*TPH2 and NΔ47‐rc*h*TPH2, binding of l‐Phe does not seem to involve the 47 N‐terminal residues. l‐Phe induced stability and shift in equilibrium toward dimer imply that TPH2 variants containing the regulatory domain are more stable as dimers.

A stabilizing effect of l‐Trp has previously been observed by McKinney *et al*. 16 who hypothesized that l‐Trp might stabilize through binding in the active site, which might tighten up flexible regions, and hereby protect the active site. The increase in *T*_m_ (DSF) upon the addition of l‐Phe or l‐Trp might, analogously to the hypothesis of McKinney *et al*. 16, occur through binding in the active site which in turn induces a more closed conformation, as it has been seen for binding of tryptophan in chicken TPH 25. However, a shift in the monomer–dimer equilibrium is only observed for l‐Phe, suggesting that the increase in *T*_m_ and half‐life occur through different mechanisms. Alternatively, l‐Phe binds in an allosteric site which stabilizes the regulatory domain through improved interactions with an adjacent monomer. Such an allosteric site in the regulatory domain has been identified in PAH, which has been found to increase PAH activity and stability and induce large conformational changes 20, 39, 40, 41. This is consistent with the binding of amino acids to ACT domains which often occurs at domain interfaces and results in conformational changes 42. This hypothesis is supported by the observed shift in monomer–dimer equilibrium and the additive effect of l‐Trp and l‐Phe observed in the DSF experiments. Such an allosteric site might have relevance *in vivo* functioning as an allosteric modulating site that stabilizes a dimer in the native tetramer (dimer of dimers). Stabilization of the tetramer is important as disruption of the native tetramer results in decreased enzymatic activity 43.

### Differential scanning calorimetry

**Figure 9:**
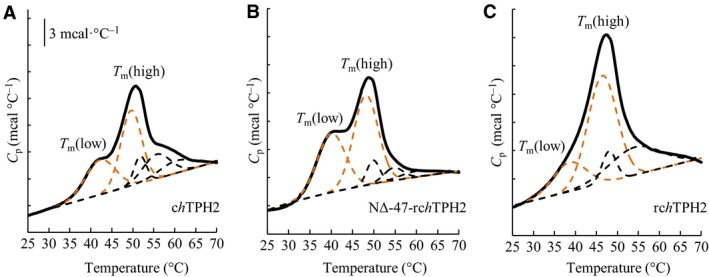
Examples of DSC thermograms of c*h* TPH2 (A), NΔ47‐rc*h* TPH2 (B), and rc*h* TPH2 (C) purified in l‐Phe buffer. All variants were analyzed at a concentration of 35 μm. DSC data after baseline subtraction are shown in solid lines and individual peaks from deconvolution as dashed lines. The quantified transitions marked *T*_m_(low) and *T*_m_(high) are highlighted in orange dashed lines.

The transition temperatures, *T*_m_, of the TPH2 variants were measured utilizing differential scanning calorimetry (DSC), to investigate if the low half‐life of *rch*TPH2 is caused by premature unfolding of the regulatory domain. The DSC experiments were performed in the presence of l‐Phe to observe domain unfolding in a monodisperse solution. All variants displayed irreversible unfolding upon reheating and hence, only *T*_m_ values were extracted from the thermograms. Illustrative examples of DSC thermograms of the three variants are presented in Fig. 9.

The unfolding transitions of the three variants seem equivalent, however, by comparison of the thermograms of c*h*TPH2 and NΔ47‐rc*h*TPH2 (Fig. 9A,B), it is evident that truncation of the regulatory domain resulted in a reduction in the heat capacity of the low temperature transition (*T*_m_(low)). This suggests that the lowest temperature transitions are partly due to the unfolding of the regulatory domain, as suggested for *h*PAH by Thórólfsson et al. 44. The remaining transitions, therefore, originate from the unfolding of the more stable catalytic domain, quantified by the transition temperature of the main peak in the thermograms (*T*_m_(high)). Transition temperatures obtained from the thermograms are presented in Table 1. Deletion of the regulatory domain increased the *T*_m_ values of the two main transitions by 1.6 and 2.9 °C, respectively. Truncation of the N terminus only induced a slight increase in the *T*_m_(high) value (*P* = 0.053, *T*‐test).

**Table 1:** The transition temperature of the investigated *h*TPH2 variants obtained from DSC, where means ± SD of *T*_m_(low) and *T*_m_(high) values (Fig. 9) obtained from three independent experiments are listed. A protein concentration of 35 μm was used in all measurements

	rc*h*TPH2	NΔ47‐rc*h*TPH2	c*h*TPH2
*T*_m_(low), °C	40.1 ± 1.2	39.6 ± 0.5	41.7 ± 0.4
*T*_m_(high), °C	46.8 ± 0.3	47.7 ± 0.5	49.7 ± 0.1

The regulatory and catalytic domains of rc*h*TPH2 unfold at 40.1 ± 1.2 °C and 46.8 ± 0.3 °C, respectively. These results are, despite variations in the buffer systems, in the same range as previously reported transition temperatures for AAAHs (47.5–55.5 °C) 33, 45, 46. The DSC data relate to the inactivation measurements, as unfolding of the variants containing the regulatory domain is initiated at around 30 °C (Fig. 9C,B), which is the temperature of the inactivation measurements, and around 35 °C for c*h*TPH2 (Fig. 9A). This might explain the significantly higher *t*_½_ values observed for c*h*TPH2 at 30 °C. Unfolding of rc*h*TPH2 shows a less profound low temperature transition compared to NΔ47‐rc*h*TPH2. This might be explained by the mobile N terminus causing a less defined regulatory domain which seems to unfold more continuously initiated at lower temperature than observed for c*h*TPH2. Furthermore, the presence of the regulatory domain causes an earlier unfolding of the catalytic domain; this signifies that attempts to stabilize TPH should occur through the regulatory domain.

### Purification yield

**Figure 10:**
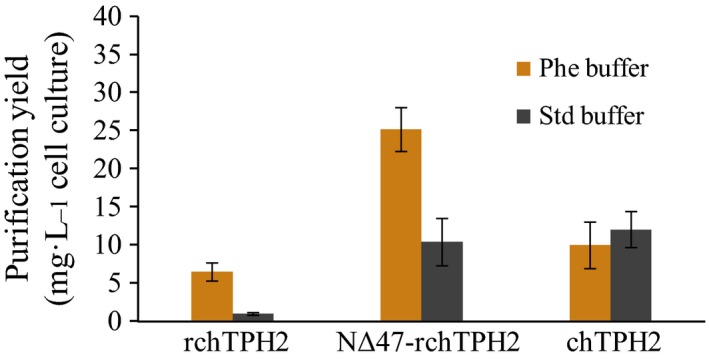
Purification yields (mg·L^−1^ cell culture, *n* = 2–6) of the three *h*TPH2 variants (mean ± SD). Orange and dark gray bars state yields obtained from purifications using Phe buffer (20 mm HEPES/NH_4_ OH, 300 mm (NH_4_)_2_ SO_4_, pH 7.0, and 3 mm l‐Phe) and Std buffer (20 mm HEPES/NH_4_ OH, 300 mm (NH_4_)_2_ SO_4_, and pH 7.0), respectively.

Characterization of *h*TPH2 variants containing the regulatory domain has been hampered by the low quantities obtained from *E. coli*, partially due to the poor stability. Figure 10 presents the purification yields obtained for the three variants with and without l‐Phe added to the purification buffer. Without l‐Phe, very low purification quantities were obtained for rc*h*TPH2 (0.9 ± 0.2 mg·L^−1^) compared to c*h*TPH2 (12.0 ± 2.4 mg·L^−1^). The low quantities for rc*h*TPH2 were overcome by truncation of the N terminus which resulted in an 11‐fold increase in yield (10.4 ± 3.1 mg·L^−1^), which was similar to that of c*h*TPH2.

The stabilizing effect of l‐Phe was reflected in the purification yields, as they were increased seven‐ and twofold for rc*h*TPH2 (6.4 ± 1.2 mg·L^−1^) and NΔ47‐rc*h*TPH2 (25.1 ± 2.9 mg·L^−1^), respectively (Fig. 10). In line with the results observed in the inactivation experiment, no significant change in purification yield was observed for c*h*TPH2 (9.9 ± 3.1 mg·L^−1^).

## Conclusion

The current results demonstrate that l‐Trp and l‐Phe change the unfolding mechanism of *h*TPH2, but only when the regulatory domain is present. Deletion of the C‐terminal tetramerization domain results in a monomer–dimer equilibrium which is shifted to predominately dimer with the addition of l‐Phe. In the presence of l‐Phe, the dimer displayed significantly increased half‐life which in turn resulted in significantly increased purification yields of *h*TPH2 variants containing the regulatory domain. These findings will facilitate future characterization of *h*TPH2.

## Materials and methods

### Materials

All used chemicals were of analytical grade, and all solutions were prepared using water from an 18.2 MΩ·cm Milli‐Q synthesis A10 Q‐Gard system which was filtered through a 0.22‐μm filter. Protein purification was performed on an ÄKTA purifier 100 from GE Healthcare. Utilized GE Healthcare column variants: HiLoad Superdex 200 26/60 pg, Superdex 200 10/300 GL, and a XK 16/20 column packed with 25 mL Dextrin Sepharose High Performance media. During purifications, all TPH‐containing solutions were kept in ice water, except during the chromatographic steps, which were performed at room temperature. Protein solutions were concentrated using an Amicon ultrafiltration cell with an Ultracell PL‐3 membrane. Protein concentrations were determined by measuring the absorbance at 280 nm on an ND‐1000 NanoDrop Spectrophotometer from Saveen Werner (Limhamn, Sweden).

### Cloning and expression

Full‐length human TPH2 cDNA optimized for expression in *E. coli* was obtained from GenScript (Piscataway, NJ, USA). All proteins were expressed as maltose binding protein fusion proteins from the pET26 expression vector in *E. coli* BL21(DE3) (Novagen, Merck Millipore, Darmstadt, Germany) cells. The sequences of the proteins expressed are given in the Supporting information. The recombinant fusion proteins contain a cleavage recognition site for human rhinovirus 3C protease (3CP) 47. The construct encoding the different protein variants was obtained by PCR. The primers used are listed in Table S1. All DNA sequences were verified by sequencing (Eurofins). Proteins were expressed at 20 °C for 14 h, as previously described 17. MBP‐3CP was cloned and expressed in‐house in a similar manner.

### Purification

*Escherichia coli* cells from 650 mL cultures were thawed from −80 °C and resuspended in buffer containing 20 mm HEPES/NH_4_OH, 300 mm (NH_4_)_2_SO_4_, pH 7.0 (standard buffer, Std buffer) or 20 mm HEPES/NH_4_OH, 300 mm (NH_4_)_2_SO_4_, pH 7.0, and 3 mm l‐Phe (Phe buffer), to a volume of 40 mL. (NH_4_)_2_SO_4_ was included in the purification buffer, as it has been found that the purification yield of recombinant catalytic core of rabbit TPH1 was enhanced by the addition of ammonium sulfate 24. The resuspended cell culture was lysed by sonication for 3 × 30 s using a Satorius Labsonic at 80% amplitude, while kept in ice water. The lysed sample was centrifuged at 4 °C and 18 000 ***g*** for 20 min. The supernatant was decanted to another tube and centrifuged a second time at 4 °C and 18 000 ***g*** for 20 min, while the pellet was discarded. The supernatant was collected and filtered through a 0.45‐μm filter. A volume of approximately 35 mL filtered supernatant was loaded with a flow rate of 5 mL·min^−1^ onto a Dextrin Sepharose column, which was equilibrated with five column volumes of Std or Phe buffer. Following sample loading, an MBP‐3CP solution was prepared by diluting MBP‐3CP from stock with Std or Phe buffer to a concentration of 2.2 μm. About 30‐mL MBP‐3CP solution was loaded onto the column with a flow rate of 5 mL·min^−1^, and the column was incubated at room temperature for 1 h. Once the flow (5 mL·min^−1^) was resumed, 10 mL of the protein‐containing eluate was collected. The collected solution, containing target protein, was filtered through a 0.45‐μm filter prior to loading on a HiLoad Superdex 200 prep grade column, which had been equilibrated with two column volumes of Std or Phe buffer. Std or Phe buffer was used as mobile phase using a flow rate of 2.5 mL·min^−1^, and UV‐detected (280 nm) peaks containing the target protein (verified by SDS/PAGE analysis – Figs S3–S5) were collected. The concentration of the collected protein was determined by UV‐Vis absorption at 280 nm utilizing theoretical extinction coefficients obtained from Expasy 48. The samples were either concentrated by ultrafiltration prior to freezing in liquid nitrogen, or if the concentration of target protein was high enough in the eluate, the ultrafiltration was bypassed, and the protein solution was frozen in liquid nitrogen and stored at −80 °C.

### SDS/PAGE

Evaluation of molecular weights and purity were conducted by SDS/PAGE. Proteins were resolved on Mini‐PROTEAN TGX gels (7.5%) from Bio‐Rad (Hercules, CA, USA) run at 100 V for 75 min with a protein standard from Bio‐Rad (no. 161‐0304). Gels were stained with Coomassie Blue to visualize the proteins.

### Differential scanning fluorimetry

The unfolding of the TPH2 variants was recorded with an Agilent Technologies Stratagene MX3005 P RT‐PCR machine (Santa Clara, CA, USA). The ligand screen was performed with a total volume of 25 μL in 96‐well plates (polypropylene plates from Agilent Technologies). Each well was composed of protein at a concentration of 1 μm, SYPRO orange at a concentration of 2× (diluted from SYPRO^®^ 5000× stock from Sigma, St. Louis, MO, USA), and ligand (diluted in purification buffer) in a concentration range of 0.1 μm to 10 mm. Each plate contained control wells with the purification buffer with and without protein and ligand. Scans were carried out using a scan rate of 1 °C·min^−1^, going from 20 °C to 95 °C. The thermograms were baseline corrected with mxpro qpcr Software (Agilent Technologies, Santa Clara, CA, USA) and analyzed for transition temperatures with graphpad prism 6 (GraphPad Software, Inc, La Jolla, CA, USA) utilizing a Boltzmann sigmoid fit:

$$y=\text{LL}+\frac{\text{UL}-\text{LL}}{1+\text{exp}\left(\frac{T_{m}-x}{a}\right)}$$

where LL and UL are the values of minimum and maximum intensities, respectively, and *a* denotes the slope of the unfolding curve at *T*_m_ 49.

### Analytical size exclusion chromatography

Determination of the oligomeric state of the TPH2 variants was performed on a Superdex 200 10/300 GL column. Prior to analysis, the column was equilibrated with two column volumes of the buffer of investigation, and the samples were spiked with concentrated stock solutions of l‐Trp, l‐Phe, or d‐Phe and allowed to equilibrate for 20 min. Samples were injected with a 500 μL loop and analyzed at a flow rate of 0.5 mL·min^−1^. Calibration curve of molecular weights was obtained from GE Healthcare 35.

The equilibrium constant of the dissociation of a dimer into monomers was based on the scheme below

$$D\overset{K_{d}}{\to }M+M$$

The equilibrium dissociation constant, *K*_d_, is defined by

(1) $$K_{d}=\frac{{\left[M\right]}^{2}}{\left[D\right]}$$

where [M] and [D] are the molar concentrations of monomer and dimer, respectively. The total protein concentration, [M]_total_, can be expressed in terms of molar monomer equivalents

(2) $${\left[M\right]}_{\text{total}}=\left[M\right]+2\left[D\right]$$

and the concentration of dimer is therefore given by

(3) $$\left[D\right]=\frac{{\left[M\right]}_{\text{total}}-\left[M\right]}{2}$$

Under the assumptions that ε_280,dimer_ = 2·ε_280,monomer_ and area under the size exclusion curve (SEC) curve (AUC) ∝ [M], the molecular concentration of monomer is given by

(4) $$\left[M\right]=\frac{\text{AUC}{}_{M}}{{\text{AUC}}_{M}+{\text{AUC}}_{D}}*{\left[M\right]}_{\text{total}}$$

where AUC_M_ and AUC_D_ are the area under the curve of the peaks representing the monomer and dimer, respectively, obtained by deconvolution of the chromatograms from the SEC experiments. Substituting [M] in equation (3) by the expression of [M] in equation (4) yields

(5) $$\left[D\right]=\frac{1}{2}\frac{{\text{AUC}}_{D}}{{\text{AUC}}_{M}+{\text{AUC}}_{D}}{\left[M\right]}_{\text{total}}$$

where AUC_D_/(AUC_M_ + AUC_D_) is the mole fraction of dimer, *f*_D_. Substituting [M] and [D] from equation (4) and (5), respectively, into equation (1) yields

(6) $$K_{d}=\frac{2*{\text{AUC}}_{M}^{2}*{\left[M\right]}_{\mathrm{total}}}{{\text{AUC}}_{D}*\left({\text{AUC}}_{D}+{\text{AUC}}_{M}\right)}$$

The sample concentration loaded in SEC will be diluted during separation. Therefore, when applying equation (6), [M]_total_ must be divided by the dilution factor, DF, introduced during gel filtration

(7) $$K_{d}=\frac{2*{\text{AUC}}_{M}^{2}\frac{{\text{[M]}}_{\text{total}}}{\text{DF}}}{{\text{AUC}}_{D}*\left({\text{AUC}}_{D}+{\text{AUC}}_{M}\right)}$$

The dilution factor during elution was measured by the width at half height of the peak divided by the sample load volume 50. This was performed only on monodisperse solutions. The fraction of dimer, *f*_D_, as a function of [M]_total_ can be expressed as

(8) $$\left[D\right]=\frac{1}{2}*f_{D}*\frac{{\left[M\right]}_{\text{total}}}{\text{DF}}$$

(9) $$\left[M\right]=\left(1-f_{D}\right)*\frac{M_{\text{total}}}{\text{DF}}$$

Substituting [M] and [D] from equation (8) and (9), respectively, into equation (1) yields

(10) $$K_{d}=\frac{{\left(\left(1-f_{D}\right)*\frac{M_{\text{total}}}{\text{DF}}\right)}^{2}}{f_{D}*\frac{1}{2}*\frac{M_{\text{total}}}{\text{DF}}}$$

Solving equation (10) for the fraction of dimer, *f*_D_, yields

(11) $$f_{D}=\frac{\frac{1}{4}\left(4*\frac{{\text{[M]}}_{\text{total}}}{\text{DF}}+K_{d}-1*\sqrt{K_{d}^{2}+8*K_{d}*\frac{{\text{[M]}}_{\text{total}}}{\text{DF}}}\right)}{\frac{{\text{[M]}}_{\text{total}}}{\text{DF}}}$$

### Activity assay

The activity measurements were performed using a Varian Cary Eclipse Fluorescence Spectrophotometer. For activity measurements, the *h*TPH2 variants were thawed under running water, filtered, and the concentrations were determined by UV‐Vis absorption at 280 nm. The *h*TPH2 samples were diluted to a protein concentration of 5 μm in the buffer in which it was purified. TPH2 activity was assayed in a reaction mixture (10 × 10 mm QS quartz cuvette from Hellma (Müllheim, Germany) – 2500 μL total volume) containing 50 mm HEPES/NH_4_OH, 200 mm (NH_4_)_2_SO_4_, pH 7.0, 0.025 g·L^−1^ catalase, 25 μm (NH_4_)_2_Fe(II)(SO_4_)_2_^.^6H_2_O, 7 mm dithiothreitol (DTT), 60 μm l‐Trp, and 300 μm BH_4_ with stirring at 15 °C 14, 51. The excitation wavelength was 300 nm, and the emission was monitored at 330 nm. The activities were determined by the initial slope (intensity·min^−1^) of the monitored fluorescence and were quantified using varian spectrophotometer software (Agilent Technologies). Quantification occurred through linear regression on a manually placed interval of minimum 0.04 min of the initial curve. Denaturation was performed by heating the protein in aliquots of 1300 μL to 30 °C in a water bath. The denaturation was stopped by cooling the protein solution in ice water.

### Differential scanning calorimetry

Differential scanning calorimetry (DSC) measurements were carried out on a TA Instruments (New Castle, DE, USA) 6300 Nano DSC. Solutions of protein in l‐Phe containing buffer were used for sample measurements, and the same buffer as used in the purification of the *h*TPH2 variants were used as references. Prior to sample loading, the protein sample and the reference buffer were degassed at approximately 10 °C using a MicroCal (MicroCal, LLC, Northhampton, MA, USA) USB ThermoVac. Three hundred microliters of protein sample and reference buffer were loaded in the respective cells. A constant pressure of 3 atm was applied, and scans were carried out using a scan rate of 1 °C·min^−1^, going from 5 °C to 70 °C. Deconvolution of the thermograms was performed with Peakfit v4.12 using Gaussian peak functions.

## Author contributions

KDT: Wrote the paper, performed experiments, and analyzed the data. HEMC and GHP: Designed experiments, contributed to data analysis and in writing the paper. NH and JB: Performed preliminary experiments. PH: Contributed to writing the paper.

## Supporting information


**Appendix S1.** Sequences of *h*TPH2 protein variants.
**Table S1.** Primers for cloning the recombinant truncated *h*TPH2 protein variants.
**Fig. S1.** Alignment of the regulatory domains of *h*TPH2 and *rn*PAH.
**Fig. S2.** Representative example of raw data from differential scanning fluorimetry.
**Fig. S3.** SDS‐PAGE results of collected peaks from purification of rc*h*TPH2 using Phe buffer.
**Fig. S4.** SDS‐PAGE results of collected peaks from purification of NΔ47‐rc*h*TPH2 using Phe buffer.
**Fig. S5.** SDS‐PAGE results of collected peaks from purification of c*h*TPH2 using Phe buffer.
