# Supplementary material for: Stabilization of tryptophan hydroxylase 2 by l‐phenylalanine‐induced dimerization
Source: FEBS Open Bio. 2016 Aug 22;6(10):987–99. doi: 10.1002/2211-5463.12100 (PMC5055035; doi:10.1002/2211-5463.12100)
Supplement: Supplementary file 1 — Appendix S1. Sequences of hTPH2 protein variants. Table S1. Primers for cloning the recombinant truncated hTPH2 protein variants. Fig. S1. Alignment of the regulatory domains of hTPH2 and rnPAH. Fig. S2. Representative example of raw data from differential scanning fluorimetry. Fig. S3. SDS‐PAGE results of collected peaks from purification of rchTPH2 using Phe buffer. Fig. S4. SDS‐PAGE results of collected peaks from purification of NΔ47‐rchTPH2 using Phe buffer. Fig. S5. SDS‐PAGE results of collected peaks from purification of chTPH2 using Phe buffer. [file FEB4-6-0987-s001.docx]

|  |
| --- |

Stabilization of Tryptophan Hydroxylase 2 by L-Phenylalanine Induced Dimerization

Kasper D. Tidemand^a^, Hans E. M. Christensen^a,b^, Niclas Hoeck^a^, Pernille Harris^a^, Jane Boesen^a^, and Günther H. Peters^a,c^

^a^Department of Chemistry, Technical University of Denmark, Kemitorvet 207, DK-2800 Kgs. Lyngby, Denmark

Corresponding authors: hemc@kemi.dtu.dk^b^, ghp@kemi.dtu.dk^c^

**Supplementary Material**

1. Sequences

Color-code:

XXXX = MBP

XXXX = 3CP

XXXX = Linker

XXXX = 3CP recognition and cleavage site (shown as ↓)

XXXX = Regulatory domain

XXXX = Catalytic domain

MBP-c*h*TPH2:

MKIEEGKLVIWINGDKGYNGLAEVGKKFEKDTGIKVTVEHPDKLEEKFPQVAATGDGPDIIFWAHDRFGGYAQSGLLAEITPDKAFQDKLYPFTWDAVRYNGKLIAYPIAVEALSLIYNKDLLPNPPKTWEEIPALDKELKAKGKSALMFNLQEPYFTWPLIAADGGYAFKYENGKYDIKDVGVDNAGAKAGLTFLVDLIKNKHMNADTDYSIAEAAFNKGETAMTINGPWAWSNIDTSKVNYGVTVLPTFKGQPSKPFVGVLSAGINAASPNKELAKEFLENYLLTDEGLEAVNKDKPLGAVALKSYEEELAKDPRIAATMENAQKGEIMPNIPQMSAFWYAVRTAVINAASGRQTVDEALKDAQTNSSSNNNNNNNNNNLGLEVLFQ↓GPEELEDVPWFPRKISELDKCSHRVLMYGSELDADHPGFKDNVYRQRRKYFVDVAMGYKYGQPIPRVEYTEEETKTWGVVFRELSKLYPTHACREYLKNFPLLTKYCGYREDNVPQLEDVSMFLKERSGFTVRPVAGYLSPRDFLAGLAYRVFHCTQYIRHGSDPLYTPEPDTCHELLGHVPLLADPKFAQFSQEIGLASLGASDEDVQKLATCYFFTIEFGLCKQEGQLRAYGAGLLSSIGELKHALSDKACVKAFDPKTTCLQECLITTFQEAYFVSESFEEAKEKMRDFAKSITRPFSVYFNPYTQSIEILKD

MBP-NΔ47-rc*h*TPH2:

MKIEEGKLVIWINGDKGYNGLAEVGKKFEKDTGIKVTVEHPDKLEEKFPQVAATGDGPDIIFWAHDRFGGYAQSGLLAEITPDKAFQDKLYPFTWDAVRYNGKLIAYPIAVEALSLIYNKDLLPNPPKTWEEIPALDKELKAKGKSALMFNLQEPYFTWPLIAADGGYAFKYENGKYDIKDVGVDNAGAKAGLTFLVDLIKNKHMNADTDYSIAEAAFNKGETAMTINGPWAWSNIDTSKVNYGVTVLPTFKGQPSKPFVGVLSAGINAASPNKELAKEFLENYLLTDEGLEAVNKDKPLGAVALKSYEEELAKDPRIAATMENAQKGEIMPNIPQMSAFWYAVRTAVINAASGRQTVDEALKDAQTNSSSNNNNNNNNNNLGLEVLFQ↓GPGNKGSSKREAATESGKTAVVFSLKNEVGGLVKALRLFQEKRVNMVHIESRKSRRRSSEVEIFVDCECGKTEFNELIQLLKFQTTIVTLNPPENIWTEEEELEDVPWFPRKISELDKCSHRVLMYGSELDADHPGFKDNVYRQRRKYFVDVAMGYKYGQPIPRVEYTEEETKTWGVVFRELSKLYPTHACREYLKNFPLLTKYCGYREDNVPQLEDVSMFLKERSGFTVRPVAGYLSPRDFLAGLAYRVFHCTQYIRHGSDPLYTPEPDTCHELLGHVPLLADPKFAQFSQEIGLASLGASDEDVQKLATCYFFTIEFGLCKQEGQLRAYGAGLLSSIGELKHALSDKACVKAFDPKTTCLQECLITTFQEAYFVSESFEEAKEKMRDFAKSITRPFSVYFNPYTQSIEILKD

MBP-rc*h*TPH2:

MKIEEGKLVIWINGDKGYNGLAEVGKKFEKDTGIKVTVEHPDKLEEKFPQVAATGDGPDIIFWAHDRFGGYAQSGLLAEITPDKAFQDKLYPFTWDAVRYNGKLIAYPIAVEALSLIYNKDLLPNPPKTWEEIPALDKELKAKGKSALMFNLQEPYFTWPLIAADGGYAFKYENGKYDIKDVGVDNAGAKAGLTFLVDLIKNKHMNADTDYSIAEAAFNKGETAMTINGPWAWSNIDTSKVNYGVTVLPTFKGQPSKPFVGVLSAGINAASPNKELAKEFLENYLLTDEGLEAVNKDKPLGAVALKSYEEELAKDPRIAATMENAQKGEIMPNIPQMSAFWYAVRTAVINAASGRQTVDEALKDAQTNSSSNNNNNNNNNNLGLEVLFQ↓GPQPAMMMFSSKYWARRGFSLDSAVPEEHQLLGSSTLNKPNSGKNDDKGNKGSSKREAATESGKTAVVFSLKNEVGGLVKALRLFQEKRVNMVHIESRKSRRRSSEVEIFVDCECGKTEFNELIQLLKFQTTIVTLNPPENIWTEEEELEDVPWFPRKISELDKCSHRVLMYGSELDADHPGFKDNVYRQRRKYFVDVAMGYKYGQPIPRVEYTEEETKTWGVVFRELSKLYPTHACREYLKNFPLLTKYCGYREDNVPQLEDVSMFLKERSGFTVRPVAGYLSPRDFLAGLAYRVFHCTQYIRHGSDPLYTPEPDTCHELLGHVPLLADPKFAQFSQEIGLASLGASDEDVQKLATCYFFTIEFGLCKQEGQLRAYGAGLLSSIGELKHALSDKACVKAFDPKTTCLQECLITTFQEAYFVSESFEEAKEKMRDFAKSITRPFSVYFNPYTQSIEILKD

MBP-3CP:

MKIEEGKLVIWINGDKGYNGLAEVGKKFEKDTGIKVTVEHPDKLEEKFPQVAATGDGPDIIFWAHDRFGGYAQSGLLAEITPDKAFQDKLYPFTWDAVRYNGKLIAYPIAVEALSLIYNKDLLPNPPKTWEEIPALDKELKAKGKSALMFNLQEPYFTWPLIAADGGYAFKYENGKYDIKDVGVDNAGAKAGLTFLVDLIKNKHMNADTDYSIAEAAFNKGETAMTINGPWAWSNIDTSKVNYGVTVLPTFKGQPSKPFVGVLSAGINAASPNKELAKEFLENYLLTDEGLEAVNKDKPLGAVALKSYEEELAKDPRIAATMENAQKGEIMPNIPQMSAFWYAVRTAVINAASGRQTVDEALKDAQTNSSSNNNNNNNNNNLGGIPGPEHEFLNALIRRNCHIITTDKGEFNLLGIYSNCAVVPTHAEPGDVVDIDGRLVRVLKQQVLTDMNDVDTEVTVLWLDQNEKFRDIRRFIPEHQQDWHNIHLATNVTKFPMLNVEVGHTVPYGEINLSGNATCRLYKYDYPTQPGQCGAVLANTGNIIGIHVGGNGRVGYAAALLRKYFAEEQ

Table S1. Primers utilized to construct the TPH2 variants.

| Variant | Forward Primer | Reverse Primer |
| --- | --- | --- |
| MBP-c*h*TPH2 | 5’- aaaagggcccgaagagctggaagatgtgc – 3’ | 5’- ttttctcgagttactaatctttcagaatttcaatgctct – 3’ |
| MBP-rc*h*TPH2 | 5’- aaaagggccccagccggcgat – 3’ | 5’- ttttctcgagttactaatctttcagaatttcaatgctct – 3’ |
| MBP-NΔ47-rc*h*TPH2 | 5’- aaaagggcccggcaacaaaggcagc – 3’ | 5’- ttttctcgagttactaatctttcagaatttcaatgctct – 3’ |
| MBP-3C Protease | 5’- aaaacatatgaaaatcgaagaaggtaaactggt – 3’ | 5’- aaaaggatcccaccgaggttgttgttattgtt – 3’ |


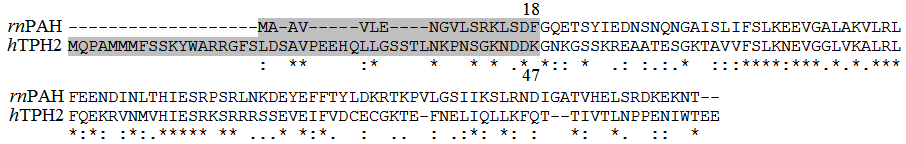


**Fig. S1. Alignment of the regulatory domains of *h*TPH2 and *rn*PAH using Clustal Omaga (1.2.1). "-" marks a gap in the primary structure. The grey background illustrates the highly mobile part of the N-terminal region, for which no crystal structure is available for *rn*PAH and the presumably equivalently mobile region of *h*TPH2. "*" marks conserved residues, ":" marks conservation of residues with highly similar properties and "." marks conservation of residues with slightly similar properties.**

Fig. S2. Representative example of raw data from differential scanning fluorimetry of rc*h*TPH2 (enzyme concentration of 1 µM) with no ligand (blue line, reference) and with addition of 10 mM L-Trp (purple line).

rc*h*TPH2:

1.
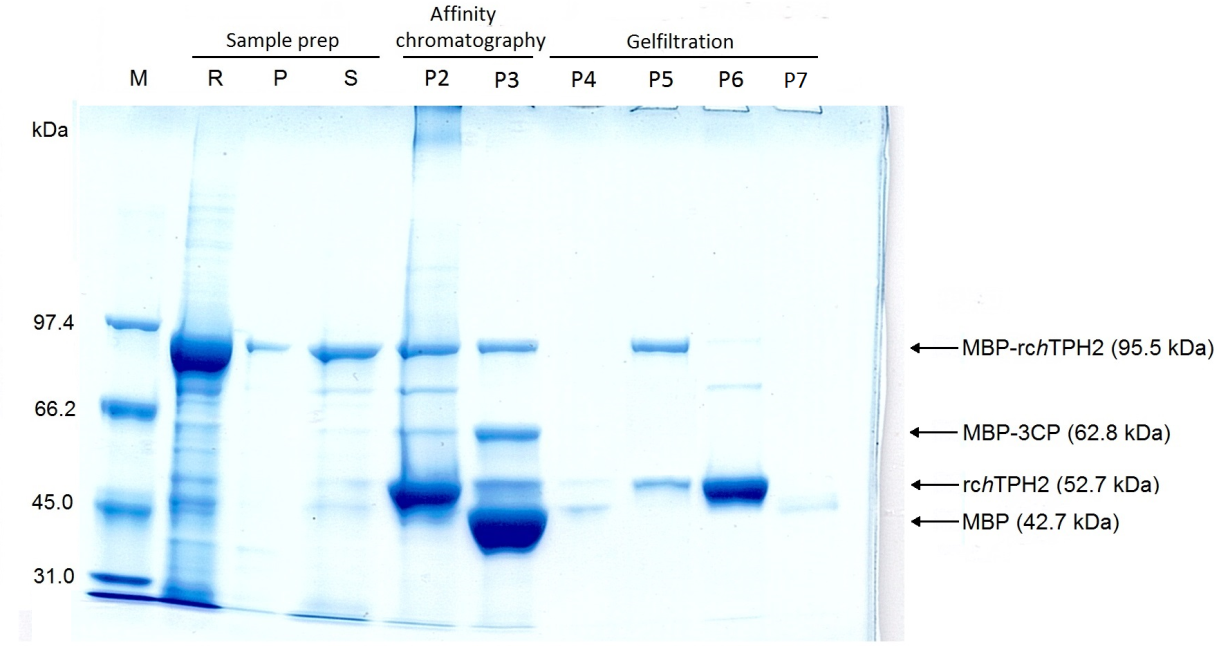


Fig. S3. SDS-PAGE results of collected peaks from purification of rc*h*TPH2 using Phe buffer. M: Molecular weight standard. R: Resuspension of cell culture. P: Resuspended pellet. S: Supernatant. P2: Peak eluting after cleavage with MBP-3CP. P3: Peak from elution of MBP-bound species. P4-P7: Peaks from size exclusion chromatography. P6: Collected fraction of rc*h*TPH2.

NΔ47-rc*h*TPH2:


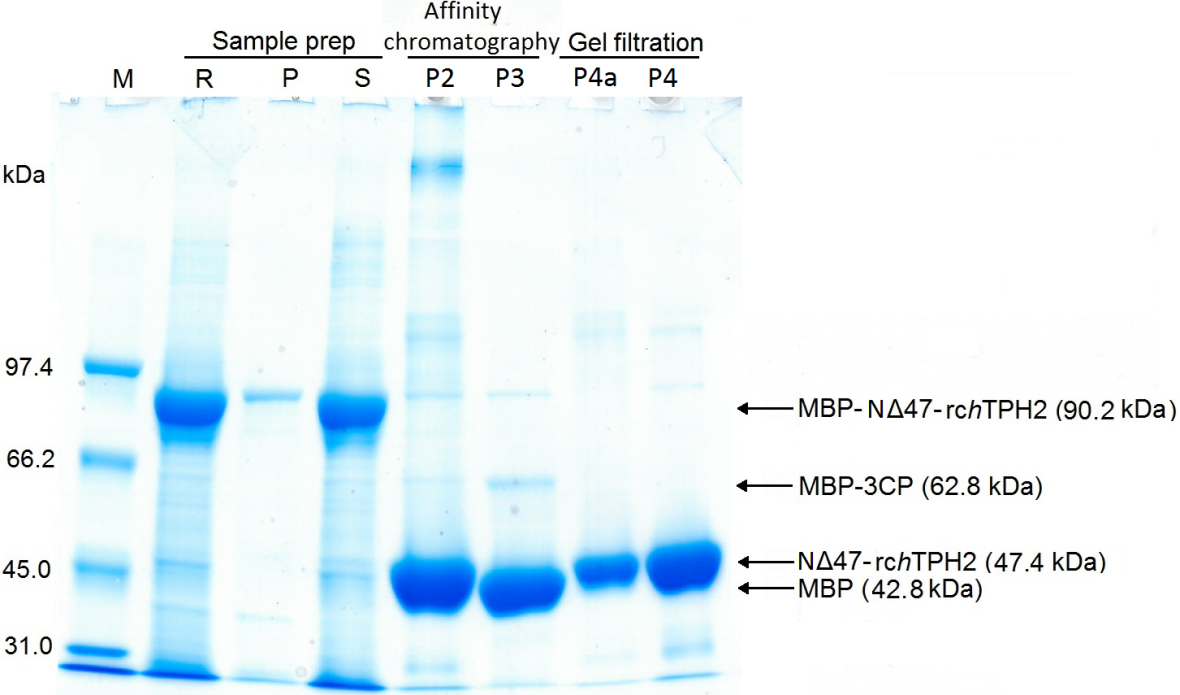


Fig. S4. SDS-PAGE results of collected peaks from purification of NΔ47-rc*h*TPH2 using Phe buffer. M: Molecular weight standard. R: Resuspension of cell culture. P: Resuspended pellet. S: Supernatant. P2: Peak eluting after cleavage with MBP-3CP. P3: Peak from elution of MBP-bound species. P4 and P4a: Peak from size exclusion chromatography. P4: Collected fraction of NΔ47-rc*h*TPH2.

c*h*TPH2:


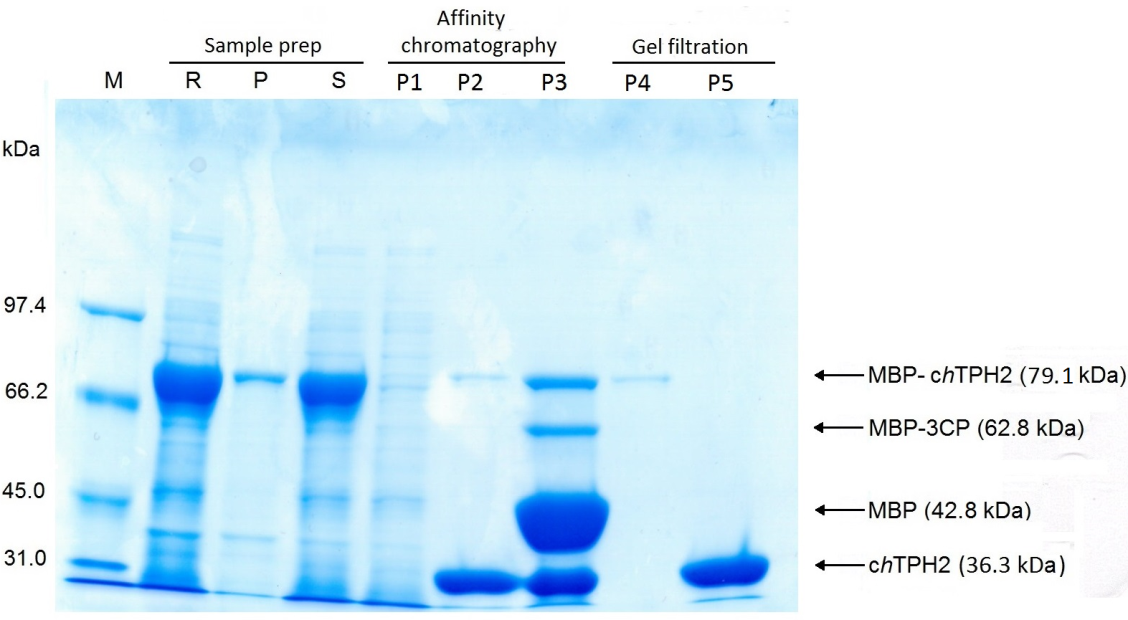


Fig. S5. SDS-PAGE results of collected peaks from purification of c*h*TPH2 using Phe buffer. M: Molecular weight standard. R: Resuspension of cell culture. P: Resuspended pellet. S: Supernatant. P1: Flow-through of unbound species. P2: Peak eluting after cleavage with MBP-3CP. P3: Peak from elution of MBP-bound species. P4 and P5: Peaks from size exclusion chromatography. P5: Collected fraction of c*h*TPH2. The presence of target protein in P3 is indicative of incomplete cleavage of 3CP protease in the on-column purification step.
